# The role of miRNAs in Behçet’s disease

**DOI:** 10.3389/fimmu.2023.1249826

**Published:** 2023-10-04

**Authors:** Feihan Gu, Xu Huang, Wenkai Huang, Mingyu Zhao, Hu Zheng, Yuanyin Wang, Ran Chen

**Affiliations:** College and Hospital of Stomatology, Anhui Medical University, Key Laboratory of Oral Diseases Research of Anhui Province, Hefei, China

**Keywords:** miRNA, Behçet’s disease, biomarker, therapy, autoimmune

## Abstract

The symptoms of Behçet’s disease (BD), a multisystemic condition with autoimmune and inflammation as hallmarks, include arthritis, recurring oral and vaginal ulcers, skin rashes and lesions, and involvement of the nervous, gastrointestinal, and vascular systems. Non-coding RNAs (ncRNAs), including microRNAs (miRNAs), long non-coding RNAs (lncRNAs), and circular RNAs (circRNAs), may be important regulators of inflammation and autoimmune disease. These ncRNAs are essential to the physiological and pathophysiological disease course, and miRNA in particular has received significant attention for its role and function in BD and its potential use as a diagnostic biomarker in recent years. Although promising as therapeutic targets, miRNAs must be studied further to fully comprehend how miRNAs in BD act biologically.

## 1 Introduction

### 1.1 Behçet’s disease

Behçet’s disease (BD) is a rare, chronic, multisystemic ailment characterized by inflammation and autoimmunity. It can cause ocular lesions, recurring oral and vaginal ulcers, skin rashes and lesions, and arthritis, and may also involve the nervous system, intestines, and blood vessels (1). Epidemiologists have found that the incidence of BD varies widely around the globe, from 0.1 per 100,000 people in Hawaii to 664 per 100,000 people in northern Jordan (2). BD is associated with several environmental factors, for instance infections and the microbiome. Viral infection has long been hypothesized to be one of the main etiological factors of BD. Infectious agents such as hemorrhagic streptococcus or differences in the composition of the saliva or intestinal microbiome trigger congenital inflammation, which is subsequently maintained by an adaptive immune response (3–5). BD has recently been classified as a form of MHC-I-opathy, and the latest research shows that the most potent genetic susceptibility factor is HLA-B51. The variable endoplasmic reticulum aminopeptidase 1 (ERAP1) haplotype Hap10 participates in the pathogenesis of BD by generating restricted HLA-B51 peptides (6–8). An epistatic interaction between HLA-B51 and ERAP1 may affect the tuning of microbial and/or endogenous peptides, disrupting the homeostasis of regulatory T cells (Tregs) and the amplification of type 1 T helper (Th1) and Th17 effector cells. Genetic differences in the expression of cytokine genes may affect their susceptibility and impact on their functionality (9). To prevent severe and potentially life-threatening consequences such as neurologic complications, the occlusion of large blood vessels, and damage to the eyes, it is crucial to detect BD in its early stages (10). Unfortunately, no specific diagnostic laboratory test exists and diagnosis relies on clinical criteria (11). Rapid diagnosis, disease activity surveillance, and therapy for BD can be greatly improved through the discovery of new and therapeutic targets and sensitive biomarkers.

### 1.2 MicroRNAs

**Figure 1:**
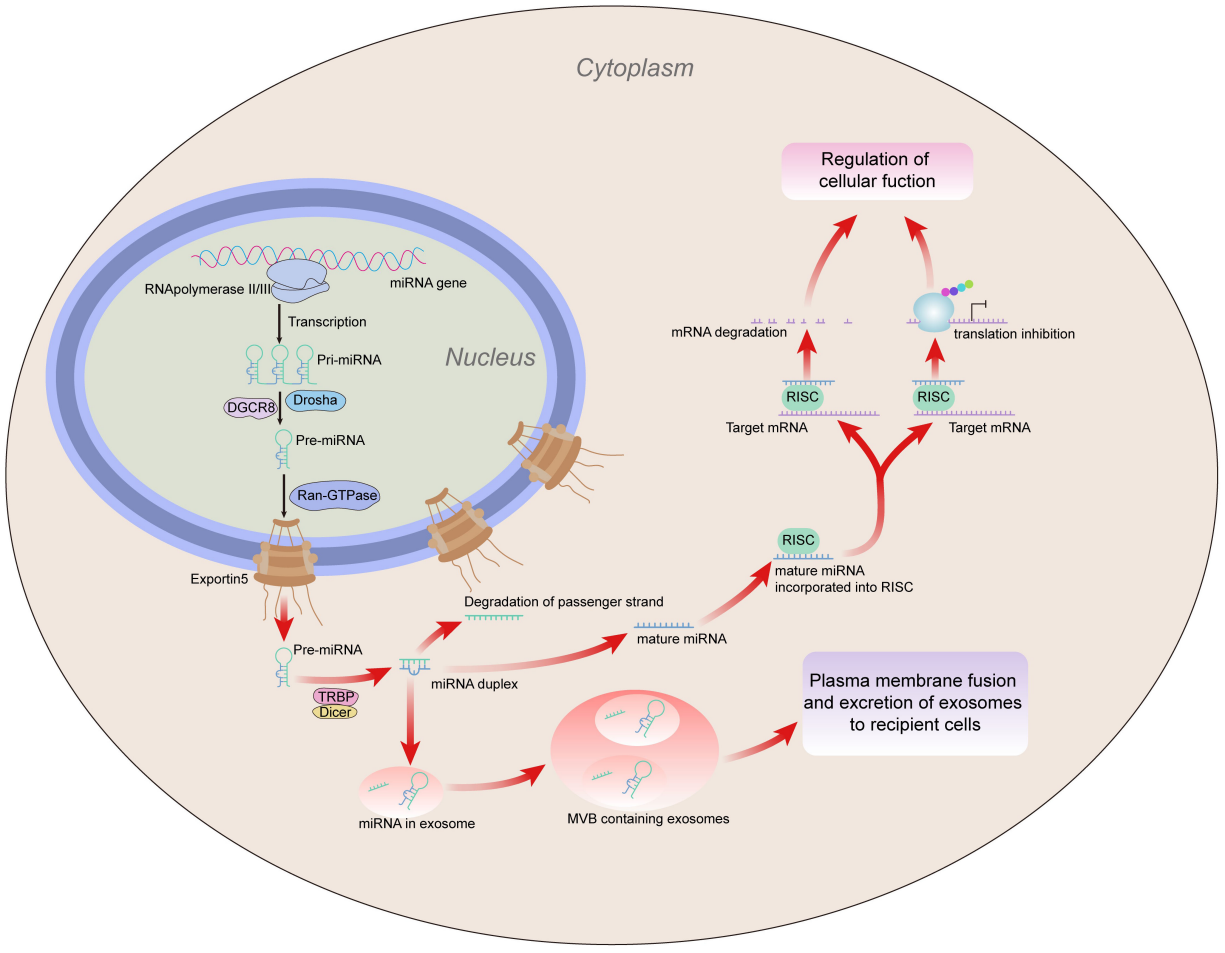
MiRNA generation. MiRNA is translated into primary (pri)miRNA by RNA polymerase (Pol) II or III, which Drosha subsequently converts into precursor (pre)miRNA. Exportin 5 moves the pre-miRNA out of the nucleus and into the cytoplasm, where Dicer converts it into a miRNA duplex. The passenger strand is destroyed during duplex unwinding, and the mature strand is incorporated into the RISC. Subsequently, the RISC regulates cell function by inhibiting the gene expression of target mRNA through mRNA degradation or inhibition of translation. In addition, miRNAs can be contained in exosomes, which are then divided into MVBs. Exosomes containing miRNA are transferred to recipient cells as a result of MVB fusion with the plasma membrane, which also acts as a mediator for intercellular gene control. miRNA, microRNA; mRNA, messenger RNA; MVBs, multivesicular bodies; RISC, RNA-induced silencing complex.

Non-coding RNAs (ncRNAs) derive from larger regions of the genome and can carry out biological functions at the RNA level but lack the ability to code for proteins (12). Through base complementary pairing, ncRNAs bind to target genes directly or indirectly and control the transcriptional translation of those genes (13, 14). Small endogenous RNAs called microRNAs (miRNAs), a subcategory of ncRNAs, control gene expression (15). More than 60% of genes that encode protein may be controlled by miRNAs (16, **Figure 1**). MiRNAs play crucial roles in immune reaction and inflammation, among other physiological and pathological processes (17–20). MiRNAs can regulate autoimmune disease BD by regulating immune cells. Type 1 T helper (Th1) cell/Th17 cell amplification and regulatory T cells (Tregs) damage has been found to be one of the main pathogenetic mechanisms of BD (21, 22); miRNAs can also participate in the pathogenesis of BD by regulating Th1 and Th17 cells, and Tregs. For example, the Tregs/Th17 cell imbalance could be caused by highly expressed miRNA-19b-3p, probably by inhibiting the expression of cluster of differentiation 46 (CD46) (23). Elevated levels of inflammatory cytokines such as interleukin-1 (IL-1) and IL-17 in BD have been found to be associated with the aberrant expression of miRNAs, such as miRNA-155, which regulates the Th17 immune response by targeting Ets-1 in BD, and miR-155 and IL-17 expression are significantly increased in CD4^+^ T cells from patients with active BD. Functional variants of miR-196a2 confer the risk of BD by regulating the expression of the miR-196a gene and regulating the production of pro-inflammatory IL-1β (24, 25). The upregulation of miR-3591-3p and downregulation of miR-638, miR-155, and miR-4488 have been linked to the etiology of BD (26–28). The role of miRNAs in BD is being investigated, and the underlying mechanism between miRNAs and BD must be clarified. Furthermore, some miRNAs may serve as potential biomarkers of illness and/or molecular tools to develop new approaches for the treatment of BD.

Research on miRNAs is critical to understanding BD. As a result, in this review we explore the function, mechanisms, and potential future applications in therapeutic research of miRNAs in BD.

## 2 Overview of BD

Most patients with BD experience oral ulcers, occurring in the buccal mucosa, gum, tongue, and lips, as their initial symptom, and often present with symptoms similar to those of recurrent aphthous stomatitis (RAS) (29). Patients with BD also commonly show symptoms of poor oral hygiene, indicated by, for example, chronic tonsillitis, periodontitis, and tooth decay (30). In patients with BD, genital ulcers are a common symptom, appearing after oral ulcers at the time of the disease. These ulcers serve as a specific clinical marker for detecting and diagnosing BD. BD skin lesions are characterized by vasculitis, which is the fundamental pathological characteristic. Furthermore, thrombophlebitis or inflammation associated with the formation of blood clots is the second most significant symptom seen in mucocutaneous BD disease. These hallmark characteristics are present not only in the skin, but also in the major organs affected by BD, such as the digestive tract, vasculature, and the central nervous system (31). In clinical practice, the choice of therapy for BD is typically made based on the patient’s clinical presentation and the organ(s) affected. To decrease the frequency/severity of episodes and to prevent consequences, the patient must be closely monitored. Although treatment is based on suppressing inflammatory reactions, the actual treatment chosen is often based on the organ(s) affected and the frequency of recurrences, severity of the involvement, duration of the disease, sex, and age at onset. BD is an inflammatory disease for which broad-spectrum, anti-inflammatory medications, biologics, immunomodulators, and immunosuppressants are available as therapeutic alternatives, because there are currently no genetic tests or diagnostic biomarkers available (32). Drugs that regulate the adaptive and innate responses to immunity, such as tumor necrosis factor alpha (TNF-α) and IL-17 blockers, have established therapeutic efficacy for the disease (33). We found that the common treatment options also have certain adverse effects (e.g., infection, iatrogenic osteoporosis, or glaucoma) (34). Thus, there is an urgent need to find new treatments. miRNAs have the possibility to serve as an alternative target for treatment. Many autoimmune diseases in humans have a correlation between their onset and prognosis and abnormal miRNA expression. Changes in miRNA expression may also play an important role in the development of BD. Therefore, miRNAs can serve as biomarkers for the diagnosis of BD or as targeted therapies for BD (35). The application of miRNA-based therapies may also achieve promising results when combined with immunotherapy, radiation, or chemotherapy. However, this area needs further research.

## 3 Functional role of miRNAs in BD

The pathogenesis of BD is related to many factors, but various studies have shown that the dysregulation of miRNA expression, such as the downregulation of microRNA 155 (miR-155), miR-23b, and miR-196 and the upregulation of miR-21 and miR-181b, may be an important cause in the pathogenesis of this disease (**Table 1**). MiRNAs are gene expression regulators involved in immune regulation. MicroRNAs are dysregulated in BD and can influence BD development and temper autoimmune reactions. A large amount of literature reporting on miRNAs and BD was found through a preliminary search of the literature for this review. However, no definitive conclusions on the functions of miRNAs in BD could be made. Therefore, the aim of this review is to list the miRNAs that have been shown to exert pathogenic mechanisms in BD, and to compare them with other MHC-I-opathy-diseases, such as ankylosing spondylitis (AS) and psoriasis, or with other autoimmune diseases. We found that the expression of miR-155 decreased in BD, but increased in AS and psoriasis, whereas the expression of miR-21 increased in BD, AS, and psoriasis.

**Table 1:** Functions of miRNAs in BD.

miRNA	Expression	Research sample	Targets and signaling pathways	Function	PMID
miR-155	−	DCs	TAB2 Akt/mTOR signaling pathway	Increase pro-inflammatory cytokines	33957967
miR-181b	+	BD patients Serum	/	Cause endothelial injury	30848985
miR-23b	−	PBMC CD4 T cells	Hes-1 Notch signaling pathway	Increase numbers of Th1/Th17 cells	24446471
miR-21	+	PBMC	RhoB _′_TLR4	Decrease cell apoptosis	25849652
miR-196	−	PBMC	Bach1/HO-1	Increase levels of pro-inflammatory cytokines	27993883

Upregulation: + ; downregulation: −

BD, Behçet’s disease; DCs, dendritic cells; PMID, PubMed identifier; PBMC, peripheral blood mononuclear cells; miRNA, microRNA; Th1 cells, T helper type 1 cells.

### 3.1 MiR-155

MiR-155 is one of the first miRNAs identified in humans, mice, and chickens. It is located on chromosome 21and its sequence is conserved (36). MiR-155 was originally defined as a B-cell integration cluster (BIC) gene that regulates adaptive and innate immunity and is encoded by the host gene *MIRHG155* (37). MiR-155 promotes dendritic cell maturation, migration to lymph nodes, T-cell activation, and cytokine release (38). Several reports have indicated that miR-155 expression is increased within a range of activated immune cells, highlighting the significant role of miR-155 in the immune response (39–41). MiR-155 is involved in the generation of cytokines by dendritic cells (DCs) induced by defective autophagy (42–44). MiR-155 may contribute to the onset of BD by regulating autophagy (45). According to one study (46), miR-155 expression is reduced in patients with active BD. As a direct target of miR-155, the transforming advancement factor-activated kinase 1 binding protein 2 (TAB2) is upregulated in the DCs of individuals with active BD and can be inhibited by miR-155 expression. TAB2 is involved in the miR-155-mediated effects on autophagy. TAB2 can interact with beclin-1 or ATG13 to control autophagy, and, in turn, affects the generation of TNF-α, IL-1, and IL-6 by DCs, which participate in the progression of BD. One study showed that *P62*/*SQSTM1*, which acts as an autophagic substrate, is decomposed during autophagy (47). The study revealed that, despite the existence of many autophagosomes in BD cells, autophagy degradation was inhibited because the levels of protein expression of *P62* in the DCs of patients with active BD were much higher than those of patients without active BD and those of healthy subjects (HSs). In summary, certain triggers in genetically susceptible individuals stimulate autophagosome formation. Therefore, the defective control of the succedent degradation of autophagy products result in local stimulation of pro-inflammatory cytokine release. MiR-155 may serve as a potential prognostic and diagnostic biomarker in BD (47). Numerous autoimmune illnesses, including rheumatoid arthritis (RA), overexpress miR-155 and are associated with a release of proinflammatory cytokines. MiR-155 expression levels were found to be significantly increased in patients with AS and in an *in vivo* model of psoriasis. Studies have shown that its upregulation leads to the increased expression of IL-21 and STAT3 as characteristic of Th-17 lymphocytes, leading to worsening inflammatory conditions in patients with AS. Through the PTEN signaling pathway, highly expressed miR-155 encourages the proliferation of psoriatic cells and inhibits apoptosis, and could be a potential therapeutic option for the treatment of psoriasis progression (48–50). Thus, the investigation of miR-155 is vital for comprehending the pathogenesis of BD. Manipulating miR-155 levels may also provide a novel approach to treating BD.

### 3.2 MiR-181b

MiR-181b belongs to the miR-181 family, of which the other members are miR-181a, miR-181c, and miR-181d (51). Based on its ability to limit the activity of activation-induced cytidine deaminases, miR-181b was identified as a regulator of the initial antibody repertoire of B cells (52). Numerous tumors and non-tumor pathologies (cardiovascular pathologies, metabolic disorders, and neurologic or infectious diseases) have been associated with levels of miR-181b (53–56). The miR-181 family, as we will describe, controls several important biological processes, for instance cell apoptosis, division, autophagy, mitochondrial properties, and immunological response (57–59). MiR-181b, the most abundant member of the miR-181 family expressed in endothelial cells (EC), has been shown to reduce EC activation by specifically targeting protein 3 *in vivo* and *in vitro.* The main site of immune-mediated damage is the endothelium (51). A key characteristic of BD is the EC damage caused by it. Growing evidence has shown that TNF-α plays an increasingly important role in the immune pathogenesis of BD, and the serum level of TNF-α is higher in patients with BD (60). The overexpression of miR-181 in BD may be a marker of endothelial injury in this disease. In one investigation, the results showed that blood levels of TNF-α, high-sensitivity C-reactive protein (hs-CRP), e-selectin, IL-6, and vascular cell adhesion molecule 1 (VCAM-1) were significantly increased in patients with BD, together with higher levels of serum miR-181b expression compared with controls (61). Importin-3 and a set of enriched NF-κB sensitive genes, including E-selectin and adhesion molecules VCAM-1, were suppressed by the overexpression of miR-181b in ECs both *in vivo* and *in vitro* (51). Conversely, Iliopoulos et al. (62) discovered that miR-181b specifically targets *CYLD* in ER-Src cells, increasing NF-κB activity and maintaining the inflammatory response. Simultaneously, Wang et al. (63) discovered that overexpression of miR-181b causes upregulated p65, a component of the NF-κB signaling pathway. This demonstrated that miR-181b targets the NF-κB signaling pathway and functions as a pro-inflammatory agent (64, 65). This miRNA-targeted therapy may be a way to modulate the inflammatory response in BD (61). However, the specific function of miR-181b in the pathological process of BD and its potential use as a diagnostic and predicted marker of the disease remains unknown, and therefore further study is needed.

### 3.3 MiR-23b

MiRNA-23b belongs to the miR-23b/27b/24-1 cluster that is encoded by the chromosomal region 9q22.32, which also generates miR-23b, a pleiotropic regulator of numerous developmental processes and clinical conditions (66, 67). Pleiotropic miR-23b plays a significant role in the control of cellular immunological responses, cell development, and cell physiological functioning (68–70). miR-23b is closely related to the appearance and progression of many diseases, including cancer and autoimmune diseases (71, 72). According to numerous studies, miR-23b modulates the pathogenesis of autoimmune diseases by targeting a variety of protein molecules, including chemokine ligand 7 (CCL-7), inhibitory-κB kinase alpha (IKK-α), the transforming advancement factor-activated kinase 1 binding protein 2 (TAB2) and the transforming advancement factor-activated kinase 1 binding protein 3 (TAB3) (73). One miRNA that is linked to the differentiation of Tregs is miR-23b. Lower levels of Tregs in BD are consistent with the lower expression of miR-23b (74). According to a recent study, miR-23b expression may be downregulated in patients with BD, which may contribute to the activation of the Notch system and of Th1/Th17 cells, and CD4 T cells transfected with the miR-23b inhibitor markedly promoted both IL-17-expressing and IFN-γ-expressing T cells. The abnormal increase in immune microenvironment stimulation and the loss of immunomodulatory effects due to the decline of miR-23b can jointly lead to the overactivation of the Notch pathway and the expansion of inflammatory cells, which may be related to the development of BD (75). Furthermore, miR-23b is considered a biomarker of RA and is negatively correlated with inflammation in RA (76). Studies have shown that inflammatory cytokines such as IL-17 promote the development inflammatory autoimmune diseases, such as systemic lupus erythematosus (SLE), whereas the suppression of miR-23b decreases the expression of the inflammatory factor IL-17 (77). Similar to RA, the inhibition of miR-23b interferes with the onset of SLE by blocking IKK-α, TAB2, and TAB3 expression. It has been predicted that the increased expression of miR-23b will aid in the treatment of SLE (73). Thus, manipulating miR-23b levels may also provide a novel approach to treating BD.

### 3.4 MiR-21

MiRNA-21 is located within the vacuolar membrane protein 1 (*VMP1*) gene on chromosome 17 (17q23.2) and is recognized as one of the most abundant and highly conserved miRNAs (78). MiR-21 expression increases in many mammalian cells and regulates various biological functions such as cell differentiation, proliferation, migration, and apoptosis (79). It also regulates many signaling pathways, and can contribute to angiogenesis, endothelial cell migration, and differentiation (80, 81). The upregulation or downregulation of miR-21 has been shown to be involved in the pathogenesis of immune diseases. Higher levels of CD4 T cells producing IL-17 were detected in BD patients (82). The differentiation of CD4 T cells in response to Th17, Th2, or Th1 may be influenced by the expressions of miR-146b, miR-21, miR-326, and their potential target transcripts (83). Serum IL-17 levels were also found to be clearly upregulated in patients with BD (84). The expression of the cytokine IL-17 has been shown to be lower in mice treated with the miR-21 antibody. RhoB and programmed cell death 4 (PDCD4) are the main functional targets of MiR-21. The expression of PDCD4 expression increases during apoptosis (85). MiR-21 exerts an antiangiogenic effect by targeting RhoB expression in endothelial cells (86). In BD mice models caused by herpes simplex virus (HSV), it was found that RhoB mRNA expression was consistently upregulated after the suppression of miR-21. According to Lu et al. (87), RhoB, PD-1, and PDCD were negatively interrelated with miR-21 expression, whereas toll-like receptor 4 (TLR4) expression was positively correlated with that of miR-21. TLR plays a crucial role in the context of the innate immune response. The inflammatory progression of BD can be explained by the accumulation of overproduced inflammatory cells and the delay of inflammatory cell apoptosis. The level of miR-21 expression has been linked to symptoms similar to those of BD-like mouse models caused by HSV. MiR-21 suppression improved BD-like inflammation symptoms by controlling cytokine (IL-6 and IL-17) expression and TLR4 levels. MiR-21 antagomir can be used as an immunomodulator to control BD-like mouse models caused by HSV (88). In addition, miR-21 is dysregulated in other immune disorders. For example, it is overexpressed in AS and psoriasis. In psoriasis, the inhibition of miR-21 by locked nucleic acid (LNA)-modified anti-miR-21 drugs reduced disease pathology in mouse xenotransplant models of patient-derived psoriatic skin. A prospective therapeutic strategy for the management of psoriasis involves targeting miR-21 (89–91). These studies will offer a potential treatment approach for individuals with BD. As immune modulators, miR-21 antagomir may be helpful for treating BD, brought on by HSV infection. The expression level of miR-21 is higher in BD patients with severe eye involvement, ROC curve analysis shows that miR-21 has a high sensitivity to the diagnosis of BD, and miR-21 can be used as a potential biomarker for the diagnosis of BD patients (83).

### 3.5 MiR-196

Hsa-microRNA-196a2 (miR-196a2), originally identified by Lagos-Quintana et al. (92), features a nucleotide change from C to T and is situated in the 3′-untranslated location of its precursor miR-196a2 (93). As a defined miRNA polymorphism, miR-196a2 is significantly associated with the risk of cancer (94, 95). Single-nucleotide polymorphisms (SNPs) are one of the most significant genetic variations in the human genome. SNPs are DNA changes that are distributed throughout human chromosomes. They are genetic variants based on single-nucleotide mutations (96). Genetic SNPs in miRNA genes may have a significant impact on how they are processed, expressed, or matured, and may lead to sensitivity to or the development of a number of diseases (97, 98). Hoffman et al. (99) demonstrated that miR-196a2 rs11614913 exerts biological effects on target gene production and also affects the transcript level of mature miR-196a. The miRNA-196a2 polymorphism is also associated with BD. Qi et al. (24) reported a higher risk of BD is conferred by the rs11614913 TT genotype in uveitis. MiR-196a2 may be a common inducer of distinct phenotypes of inflammatory bowel disease (IBD) and BD. It has been reported that a functional variant of miR-196a2 increases the risk of BD in uveitis by controlling the expression of the *miR-196a* gene. The current findings demonstrate that miR-196a2 expression is reduced in patients with active intestinal BD. Downregulated miR-196a2 may be participated in intestinal BD pathogenesis by targeting Bach1/*HO-1*. Lower expression of miR-196a2 downregulates *HO-1* gene expression and enhances the expression of the pro-inflammatory cytokines IL-17, IFN-γ, TNF-α, and IL-1β in monocytes, which may be related to the pathogenesis of intestinal BD (100). There is also research that shows a functional variant of miR-196a2 confers risk for BD, but not for Vogt–Koyanagi–Harada (VKH) syndrome or acute anterior uveitis (AAU), by modulating the expression of the *miR-196a* gene and regulating the production of pro-inflammatory IL-1β and monocyte chemoattractant protein-1 (MCP-1) (24).

Other miRNAs also play a significant role in BD. Research shows that patients with BD have impaired regulation of the inflammatory state, which could be caused by abnormal T-cell homeostasis. The upregulation of the Th17 and Th1 pathways and the reduction in the suppressive regulation of Treg cells may play vital roles in this scenario. A study by De Santis et al. found that the increased expression of miR-106b-25 may affect Tregs by altering the TGF-β signaling pathway (101). The differentiation of CD4 T cells into Th17, Th2, or Th1 responses may be influenced by the expression of miR-146b and its potential target genes (102). MiR-326 plays a key role in regulating Th17 differentiation and thus contributes to the pathogenesis of BD. The association between the lack of vitamin D and the pathogenesis of BD is generally known. A higher expression level of the *miR-326* gene inhibits the expression of VDR. MiR-326 mediates Th17 differentiation by inhibiting Ets-1 translation and is a negative regulator of Th17 differentiation. Thus, miR-326 can be considered a therapeutic target (103). MiR-326 shows high sensitivity and specificity in the prediction of ocular involvement and uveitis in patients with BD. Measuring the expression rate of miR-326 can be used as a biomarker to predict severe ocular involvement and uveitis (83).

## 4 Regulatory mechanism of miRNAs in BD

**Figure 2:**
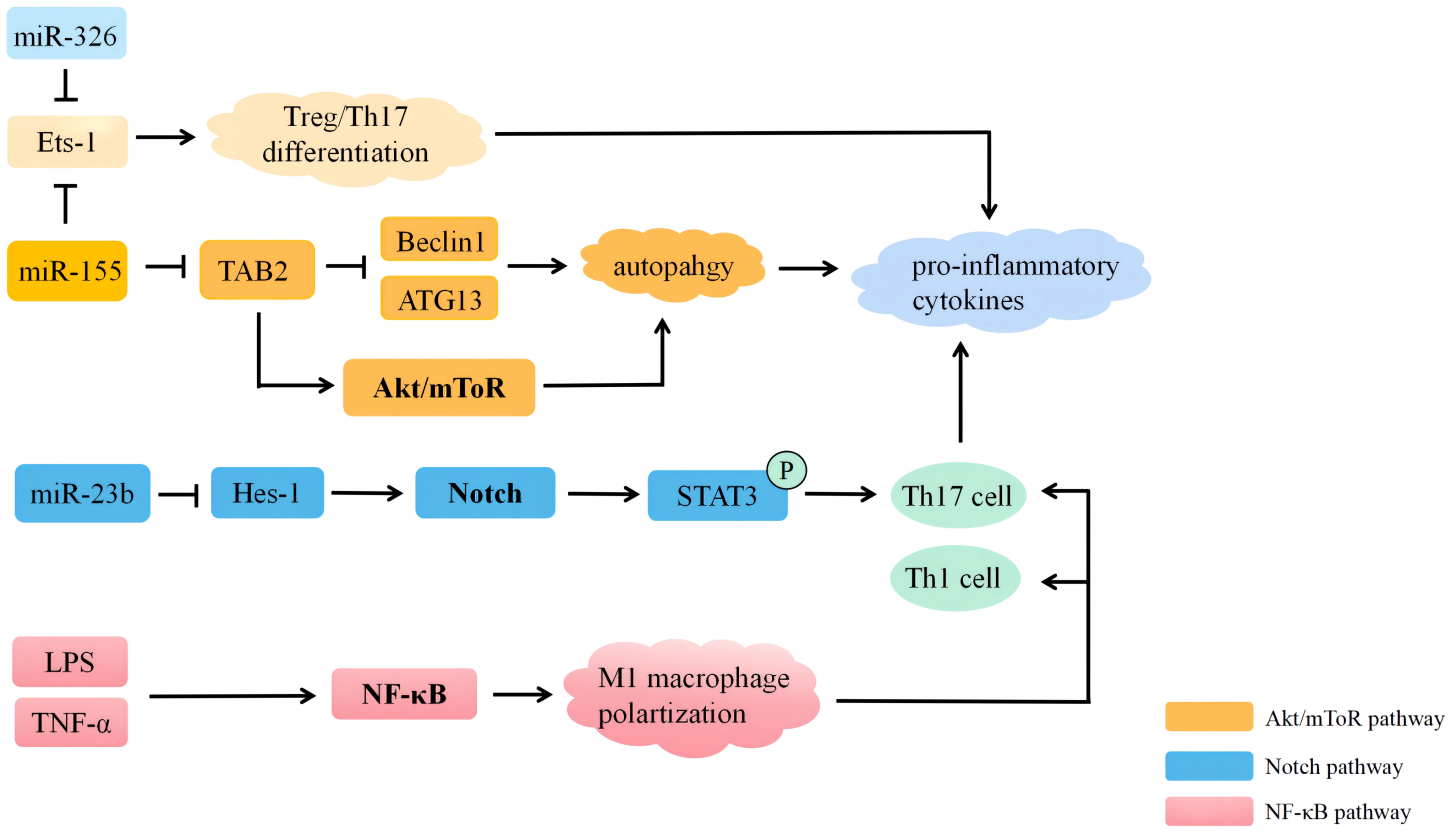
MiRNAs participate in the regulation of Behçet’s disease through multiple signaling pathways. MiRNA, microRNA; LPS, lipopolysaccharide; ATG13, autophagy-related 13; Tts-1, E26 transformation-specific-1; TAB2, the transforming advancement factor-activated kinase 1 binding protein 2; Hes-1, hairy and enhancer of split 1.

The pathogenesis of BD is complex and involves the activation and dysregulation of multiple signaling pathways, including the Notch signaling pathway, the phosphatidylinositol 3-kinase (PI3K)/protein kinase B (AKT) signaling pathway, and the nuclear factor kappa B (NF-κB) signaling pathway. Moreover, different miRNAs can regulate the development of BD by mediating these signaling pathways. Recent studies have shown that the pathogenesis of BD mediated by miRNA includes autophagy and apoptosis. Therefore, mechanisms involved in miRNA regulation interact with each other and influence the development of BD together. Exploring the pathogenesis of BD mediated by miRNA may allow us to discover novel approaches to treat patients with BD (**Figure 2**).

### 4.1 Notch signaling pathway

The Notch signaling pathway is a highly conserved pathway in mammals and is essential for immune cell homeostasis and differentiation (104). It is also intimately connected to the transmission of immunological signals. The Notch signaling pathway has no significant pro-inflammatory or anti-inflammatory effects, although its effects are greatly determined by the type of immune cell and the cellular environment (105). A previous study reported that the enhanced activation of the Notch signaling pathway may be strongly related to the etiology and pathogenesis of BD. Hes and Hey proteins are transcriptional repressors that negatively control downstream target gene expression (106). *Hes1* is the most representative target gene in the Notch signaling pathway, and *Hes1* mRNA expression is upregulated in the peripheral blood mononuclear cells (PBMCs) of patients with BD, indicating increased activation of the Notch signaling pathway, which may result in increased STAT3 phosphorylation and, in turn, activation of the Th17 response. This pathway may play a role in BD lesions. Several miRNAs have been shown to regulate the Notch signaling pathway. The activation of the Notch signaling pathway in BD may be related to the reduced expression of miR-23b (75). In patients with BD, the reduced expression of miR-23b may help to activate and amplify the function of Th17 and Th1 cells and the Notch signaling pathway. The Th17 response can be preferentially suppressed by blocking the Notch signaling pathway. Studies have shown that members of the NF-κB family control miR-23b transcription, and that this control is based on the Act-1/IL-17 signaling pathway, which implies that there is a vicious cycle among miR-23b, the Notch signaling pathway, and IL-17 (73).

### 4.2 AKT/mammalian target of rapamycin signaling pathway

The AKT/mammalian target of rapamycin (mTOR) signaling pathway regulates many cellular processes, including cellular proliferation, metabolism, and viability. The PI3K/AKT/mTOR signaling pathway is generally present in all tissues and cells (107). It plays a significant role in proliferation, cell autophagy, apoptosis cell cycle, and differentiation. One of the most well-known signaling mechanisms controlling autophagy is the Akt/mTOR signaling pathway (106, 108). In DCs, miR-155 can downregulate the phosphorylation of Akt and mTOR. There are reports indicating that, following the transfection of miR-155 mimics, the p-Akt/Akt and p-mTOR/mTOR ratios in the DCs treated with LPS increased markedly (45). MiR-155 may induce the Akt/mTOR signaling pathway to initiate autophagy and may also participate in the development of BD (27, 109). Patients with active BD have activated autophagy in their DC, and TAB2 mRNA can not only translate proteins to keep TLR4 signaling activated [the interaction of which with the gut microbiota may be involved in the development of BD (110)] but also compete with miR-155 to enhance the expression of the LPS-stimulated SOCS1 protein to prevent extensive immune responses (111). The regulation of autophagy by the AKT/mTOR pathway may be related to the pathogenesis of BD.

### 4.3 NF-κB signaling pathway

The NF-κB signaling pathway is a key regulator of cell survival, immunity, inflammation, carcinogenesis, and organogenesis (112). Once activated, the NF-κB signaling pathway regulates a number of transcriptional outcomes, driving the production of proteins essential for cell survival, cell cycle progression, immunological control, and NF-κB regulators, establishing a negative feedback loop (113). The NF-κB signaling pathway is crucial to the pathogenesis of BD. Numerous autoimmune disorders, such as SLE, RA, and IBD, have been linked to an imbalanced macrophage polarization (114). Studies have shown that BD serum promotes macrophage polarization toward the pro-inflammatory phenotype of M1 macrophage through NF-κB signaling. Thus, enhanced phagocytosis and M1 macrophage polarization may play a role in the inflammation induced by BD through their promotion of Th1 and Th17 differentiation (115). In a recent study, the phosphorylation of p65 and degradation of IκBα, and also the upregulation of TNF-α in macrophages treated with BD serum, demonstrate the activation of the NF-κB signaling pathway, leading to its amplification. Inducing NF-κB activation in BD can result in a higher profile of inflammatory cytokines, which in turn creates an environment polarized by M1 (38). Other studies have shown that NF-κB regulates genes involved in inflammatory responses and genes associated with inhibiting apoptosis, leading to resistance to apoptosis in the T-cell subpopulations of BD and participation in the pathogenesis of BD. The apoptotic refractoriness of activated T cells in BD is what leads to a prolonged inflammatory response. Key antiapoptotic proteins, the short isoform of Bcl-xL and cellular FLIP (cFLIP), were significantly upregulated in activated T cells in BD. The apoptosis-refractory properties of activated T cells in BD are what trigger the inflammatory response (116). Overall, NF-κB is crucial to the pathogenesis of BD, and inhibiting the NF-κB signaling pathway or using miRNA-targeted therapy may be effective ways to reduce the inflammatory response in BD (65).

## 5 Future expectations

The pathogenesis of BD is still unclear, and there is no unique clinical feature or laboratory diagnostic test for this disease; thus, its diagnosis often relies on clinical symptoms. However, the course of BD is typically very prolonged, and it may require months or years for all of the typical signs and symptoms to manifest (117). Early and accurate diagnosis can reduce the pain experienced by and the mortality rate of patients with BD (118). Thus, there is an urgent need for the development of an accurate detection tool supporting clinical diagnosis. To date, endogenous proteins and metabolites have been thoroughly analyzed using new “omics” approaches, such as proteomics and metabolomics. Due to the exceptional sensitivity of metabolomics, it is possible to test for changes in physiological and pathological metabolites in the early stages of a disease. Serologic markers and urinary tissues play an important role in the diagnostic process of autoimmune diseases. Zheng et al. (119, 120), taking advantage of the rise in “precision medicine” in defining a cure, determined that treating patients with disease-specific biomarkers facilitates early diagnosis and treatment modification. There is growing evidence that miRNAs play a key role in Behçet’s disease, and, because miRNAs can be easily tested in multiple biological samples, including serum, tissue, and other body fluids, they have become a suitable biological source for pathogenic research and the development of disease biomarkers (121). Using a chip, Giacomo et al. (122) evaluated ci-miRNA profiles from a screening cohort consisting of 16 patients with BD and 18 healthy controls, 29 human circulating MRNAs (ci-miRNAs) that were deregulated (DE, differentially expressed) were discovered and subsequently verified using a poly(T) adaptor PCR (PTA-PCR). Three combinations of human ci-miRNAs (hsa-miR-224-5p, hsa-miR-206, and hsa-miR-653-5p) have emerged from the latter to distinguish patients with BD from those with HC, and this combination may also help in the differentiation of patients with BD from those with HC, between patients with active BD and inactive BD, and also of BD patients from those with giant cell arteritis (GCA) and SLE. The ci-miRNA profile A may represent a new candidate biomarker that may be applied to design new therapeutic approaches for minimally intrusive BD.

Recently, advances in body fluid biopsies have reduced invasiveness, improved testing times, and achieved greater patient acceptance. MiRNAs are stable in bodily fluids (e.g., plasma, serum, and urine) and exosomes. Therefore, accurate miRNA profiling of biological fluids is attractive in clinical translational studies for the development of biomarkers and diagnostics (123). Ahmadi et al. (124) isolated PBMCs by taking blood samples from 58 healthy controls and 47 patients with BD. Using flow cytometry, the frequency of Tregs and Th17 was evaluated. A real-time PCR test was used to measure the transcription factors specific to these cells and the miRNAs that target them. Patients with BD had higher levels of Th17-associated cytokines in the cellular supernatant. Compared with healthy controls, the expression of T-cell-associated miRNAs miR-326, miR-25, miR-106b, and miR-93 increased dramatically, whereas the levels of miR-146a and miR-155 decreased, in PBMCs of BD patients. Therefore, the analysis of immune cells and their associated miRNA profiles can be used as both a therapeutic strategy and a prognostic biomarker in the treatment of patients with BD. The expression of 750 mature human miRNAs in the PBMCs of five patients with BD was compared with those of three patients with HC by Erre et al. (125), who utilized TaqMan Low Density array-based microRNA expression profiling. It was found that 13 miRNAs showed differences between BD and HC patients. Among them, miR-720 and miR-139-3p were markedly increased in the PBMCs of BD patients, which was validated by qRT-PCR. Thus, miR-720 and miR-139-3p deserve further validation as biomarkers of BD in larger studies. Currently, there is a lack of clinical applications for miRNAs, and the findings of the existing studies are based on a small sample of BD patients. The results from different patient groups have differed significantly. Nevertheless, miRNAs remain highly attractive, straightforward, and precise biomarkers, particularly for BD.

Targeting these dysregulated endogenous miRNAs may serve as a promising therapeutic strategy. However, miRNA expression profiles are influenced by many factors at the cellular level, including the microenvironment. It should be noted that there are challenges associated with the use of miRNA-based treatments in clinical practice. For example, miRNAs are prone to degradation and loss of efficacy in lysosomes. This problem can be solved by constructing more stable delivery systems, such as constructing nanoparticles that contain miRNA loads. To deliver miR-125a to splenic T cells, Zhang et al. (126) developed the monomethoxy (polyethylene glycol)-poly (d, L-lallide-co-ethanolide)-poly (L-lysine) (mPEG-PLGA-PLL) nanodelivery system. Loading miR-125a, the mPEG-PLGA-PLL (PEALmiR-125a) nanoparticle (NP) has excellent biocompatibility and can prevent miR-125a decomposition and extend the miRNA cycle time in the body. PEALmiR-125a NP therapy significantly alleviated the progression of SLE by reversing effector/Tregs imbalances. SLE is also an autoimmune disease with a pathogenesis similar to that of BD as an effector. Unfortunately, at present, there have been no reports on the effects of miRNA-related nanoparticles on Behçet’s disease; this area could be a focus for future studies. Previous studies have evaluated similar therapeutic approaches that use RNA-loaded nanoparticles to specifically silence gene expression for the treatment of numerous disorders [RNA interference (RNAi) mediated by small interfering RNAs (siRNAs) is a potent technique], such as cancer, viral infections, and autoimmune diseases. siRNA was used to reduce the *in vitro* overexpression of TNFα in a mouse model of BD to improve chronic inflammation (127). Studies have shown that although siRNA has therapeutic potential, it is challenging to deliver to target cells due to its poor stability in physiological fluids. Therefore, siRNA delivery systems such as cationic liposomes, lipids, and polymers have been developed to improve the therapeutic efficacy of siRNA. Polymerized siRNA nanocomplexes (poly-siRNA) targeting TNF-α with the mercaptoglycol-chitosan (tGC) polymer have been designed for the treatment of RA (128). The use of psi-tGC-NP therapy for specific cytokines represents a new therapeutic approach for the treatment of BD, and future research is needed in this field. However, evidence supporting the practical application of miRNA calls for more clinical trials in the future. It is fascinating that combination approaches exploiting different techniques could be developed to achieve enhanced efficacy. The rapid exploitation of these multidisciplinary drug delivery systems has identified targets for the treatment of BD by effectively regulating miRNA activity.

## 6 Conclusion

Through the in-depth study of miRNA, many new discoveries have been made about the pathobiology of BD. In BD patients, miRNA dysregulation may result in abnormal levels of inflammatory and immune-suppressive cells and cytokines. However, the regulatory pathways of miRNA-mRNA in BD remain unclear, and, to fully understand the molecular mechanisms of miRNA and BD, much work still needs to be carried out. This review summarizes the results of studies that have investigated several important miRNAs, and also the effects of miRNAs on various signaling pathways, and may therefore provide a basis for future studies. Early diagnosis using miRNAs as biomarkers can assist clinicians in their treatment, avoid occasionally inappropriate treatments and delayed diagnoses. The critical roles of miRNAs in BD cannot be ignored; nonetheless, the molecular mechanisms underlying BD still need to be clarified. Thus, additional animal models and *in vitro* studies are required. Patients with BD may benefit greatly if scholars can overcome some of the challenges associated with regulating these miRNAs.

## Author contributions

FG and XH designed the project and wrote the manuscript. WH, MZ, and HZ conducted the collection and/or assembly of data, data analysis, and interpretation. YW and RC gave final approval of manuscript and financial support. All authors contributed to the article and approved the submitted version.
